# Education on Depression in Mental Health Apps: Systematic Assessment of Characteristics and Adherence to Evidence-Based Guidelines

**DOI:** 10.2196/28942

**Published:** 2022-03-09

**Authors:** Laura Martinengo, Anne-Claire Stona, Lorainne Tudor Car, Jimmy Lee, Konstadina Griva, Josip Car

**Affiliations:** 1 Centre for Population Health Sciences Lee Kong Chian School of Medicine Nanyang Technological University Singapore Singapore Singapore; 2 Family Medicine and Primary Care Lee Kong Chian School of Medicine Nanyang Technological University Singapore Singapore Singapore; 3 Department of Primary Care and Public Health School of Public Health Imperial College London London United Kingdom; 4 Department of Psychosis & North Region Institute of Mental Health Singapore Singapore; 5 Neuroscience and Mental Health Lee Kong Chian School of Medicine Nanyang Technological University Singapore Singapore Singapore

**Keywords:** health literacy, mental health literacy, depression, mobile apps, apps, telemedicine, mHealth, self-management, mobile phone

## Abstract

**Background:**

Suboptimal understanding of depression and mental health disorders by the general population is an important contributor to the wide treatment gap in depression. Mental health literacy encompasses knowledge and beliefs about mental disorders and supports their recognition, management, and prevention. Besides knowledge improvement, psychoeducational interventions reduce symptoms of depression, enhance help-seeking behavior, and decrease stigma. Mental health apps often offer educational content, but the trustworthiness of the included information is unclear.

**Objective:**

The aim of this study is to systematically evaluate adherence to clinical guidelines on depression of the information offered by mental health apps available in major commercial app stores.

**Methods:**

A systematic assessment of the educational content regarding depression in the apps available in the Apple App Store and Google Play was conducted in July 2020. A systematic search for apps published or updated since January 2019 was performed using 42matters. Apps meeting the inclusion criteria were downloaded and assessed using two smartphones: an iPhone 7 (iOS version 14.0.1) and a Sony XPERIA XZs (Android version 8.0.0). The 156-question assessment checklist comprised general characteristics of apps, appraisal of 38 educational topics and their adherence to evidence-based clinical guidelines, as well as technical aspects and quality assurance. The results were tabulated and reported as a narrative review, using descriptive statistics.

**Results:**

The app search retrieved 2218 apps, of which 58 were included in the analysis (Android apps: n=29, 50%; iOS apps: n=29, 50%). Of the 58 included apps, 37 (64%) apps offered educational content within a more comprehensive depression or mental health management app. Moreover, 21% (12/58) of apps provided non–evidence-based information. Furthermore, 88% (51/58) of apps included up to 20 of the educational topics, the common ones being listing the symptoms of depression (52/58, 90%) and available treatments (48/58, 83%), particularly psychotherapy. Depression-associated stigma was mentioned by 38% (22/58) of the apps, whereas suicide risk was mentioned by 71% (41/58), generally as an item in a list of symptoms. Of the 58 included apps, 44 (76%) highlighted the importance of help seeking, 29 (50%) emphasized the importance of involving the user’s support network. In addition, 52% (30/58) of apps referenced their content, and 17% (10/58) included advertisements.

**Conclusions:**

Information in mental health and depression apps is often brief and incomplete, with 1 in 5 apps providing non–evidence-based information. Given the unmet needs and stigma associated with the disease, it is imperative that apps seize the opportunity to offer quality, evidence-based education or point the users to relevant resources. A multistakeholder consensus on a more stringent development and publication process for mental health apps is essential.

## Introduction

### Background

Depression affects >264 million people worldwide and was the third major contributor of years lost to disability in 2017 [1]. Yet, approximately 50% of the people living in high-income countries and at least 75% of the people living in low- and middle-income countries [2,3] have neither been diagnosed nor receive treatment. Untreated depression is associated with increased morbidity and mortality, including death by suicide, poverty and unemployment at an individual level, and significant increase in health expenditure at health system level because of increased health care use and decreased workforce productivity of the affected individuals [3,4]. Although the reasons for this wide treatment gap are multifarious, suboptimal understanding of depression and mental health disorders in the general population is considered an important contributor to low use of mental health services. Studies assessing the general population’s mental health literacy through the use of standardized clinical vignettes have shown poor recognition of common mental health disorders [5-7].

### Mental Health Literacy

Mental health literacy is defined as “the knowledge and beliefs about mental disorders, which aid their recognition, management or prevention” [8]. It encompasses the acquisition of factual knowledge of mental health disorders as well as development of competencies and beliefs enabling prevention, early recognition of symptoms, help seeking, self-help, and provision of first aid to others [5]. Evidence-based clinical guidelines for management of depression indicate that psychoeducation is pivotal in terms of disease management [9-12] and necessary to expand the shared-decision model of care in mental health services [13].

### Educational Topics

Educational topics focused on depression may include information related to its natural history, symptoms and signs, treatment options such as pharmacotherapy and a range of nonpharmacological treatments and possible side effects, as well as information related to prognosis and effective self-management interventions [9]. Psychoeducational interventions have been shown to increase mental health literacy and, most importantly, to yield small but significant reductions in symptoms of depression and mental distress [14,15], hence offering simple, inexpensive, and readily available tools for symptom management [14].

There are other notable benefits of psychoeducation, particularly stigma reduction [16-18] and increased help-seeking behavior [6,19-21]. Mental health stigma remains a significant barrier to the use of mental health services because it affects the access and quality of health care provision for people living with mental health disorders and depression [22,23]. Social stigma or public stigma are linked to discrimination, avoidance, and inadequate treatment [24], whereas self-stigma or internalized stigma may erode self-esteem and self-efficacy or lead to anger or indifference [24], which may in turn hamper help seeking [25]. Although multilevel coordinated and sustained efforts are needed to mitigate the prevailing mental health stigma, simple interventions such as the use of personal narratives have shown promise in fostering depression awareness [26,27] and more positive attitudes among the public [26,28,29].

### Mental Health Apps

Since the early 2000s, the search for health information has shifted increasingly to the web [30], and this trend has further intensified with the advent of smartphones [31] and mobile apps. A recent systematic review on the provision of medical education using smartphones reported that approximately two-thirds of the reviewed interventions were effective in improving patients’ knowledge and clinical outcomes [32]. The Apple App Store and Google Play, which are the major sources of apps worldwide [33], currently include >10,000 mental health apps [34] that offer a wide range of functionalities comprising education, screening, and self-management programs for a wide range of disorders [35,36]. However, most of the published apps have not been assessed in clinical trials and are not evidence based [34,37].

Given the increasing popularity of health apps, a number of studies evaluating direct-to-consumer apps have been published in recent years, including the general characteristics of the apps [38], key features and functionalities of highly downloaded [39] or highly rated apps [40], techniques commonly used in face-to-face psychotherapy [41], or adherence of self-guided app interventions to evidence-based clinical guidelines [42]. These studies include a variety of assessment methodologies, from researcher-developed checklists [38,41,42] used in specific projects to the use of standardized assessment tools such as the Mobile App Rating Scale [36,43] or the Organization for the Review of Care and Health Applications–24 Question Assessment [44], focusing on the usability and technical aspects of the apps. In contrast, the health-related content of apps is seldom included in assessment checklists.

Existing mental health apps often include educational components, either as its sole functionality or within a depression or mental health management app. The trustworthiness, adherence to evidence, and depth of information offered by these apps is unclear because, to date, no reviews offering an in-depth assessment of the content of education on depression modules provided by mental health apps has been published.

Therefore, this study aims to systematically evaluate the adherence to evidence-based clinical guidelines of the information on depression provided by mental health and depression apps available in the Apple App Store and Google Play.

## Methods

We followed a rigorous assessment process, developed at our center and used in previous app assessment projects [45-48], by adapting systematic review methodology for the app search, selection, assessment, and data analysis.

### Development of the Assessment Criteria

The research team designed the assessment criteria to evaluate the clinical and technical features of apps. The criteria included the following three domains:

1. *General attributes,* as described in the app store description, including developer, category, ratings, education-delivery format, target group, country of origin, and cost.
2. *Appraisal of depression education modules,* comprising the scope of information provided by the app and its adherence to evidence-based clinical guidelines from the Royal Australian and New Zealand College of Psychiatrists [11], Singapore’s Ministry of Health [49], the United Kingdom’s National Institute for Health and Care Excellence [50], and the American Psychiatric Association [9], as well as concordance with expert opinions on content of mental health awareness public campaigns [51,52]. The criteria included 46 questions encompassing 38 educational topics on symptoms and diagnostic criteria of depression, natural history, importance of help seeking, treatment (including pharmacological and nonpharmacological treatments, side effects of medication, and importance of treatment adherence), stigma, recovery, and suicide prevention information and resources. Multimedia Appendix 1 [9,11,49,50,53] presents the educational content appraisal questionnaire.
3. *Technical aspects and quality assurance of the app,* including usability, app credibility, in-app advertisements, privacy and security safeguards, and gamification.

### Selection of Apps

The Apple App Store and Google Play were systematically searched using 42matters, a proprietary app database [54], on July 8, 2020, using the terms *depression*, *depressive*, *depressed*, *mood disorder*, *sadness*, and *melancholia*. The search was limited to four app store categories: education, health and fitness, lifestyle, and medical. Eligible apps were required to conform to the criteria presented in Textbox 1.

**Figure 1:**
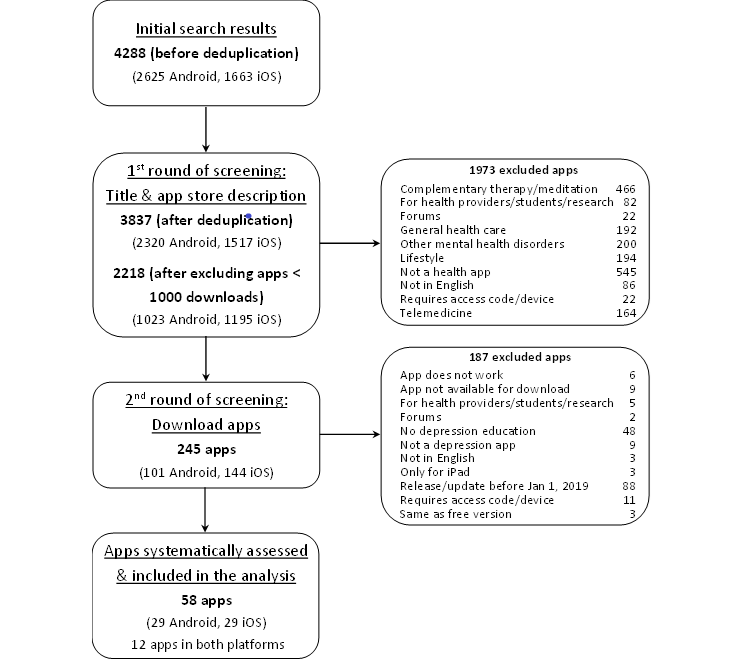
App selection flowchart.

The app selection process is presented as a flowchart (Figure 1) [55]. Before screening for eligibility, we excluded all Android apps with <1000 downloads as reported in the 42matters search output. As iOS does not report the number of app downloads, the iOS versions of the excluded Android apps were also excluded on the assumption that the app would have a similar number of downloads in both app stores. The remaining apps underwent a 2-step selection process that consisted of (1) screening the app name and app store description from the 42 matters search output and (2) downloading and screening for eligibility all apps included in step 1. Working in parallel, 2 pairs of investigators (LM and Goh Jun Wei; LM and Matthew Teo Siu Yan) independently completed the app selection process. Disagreements were resolved through discussion.

**Inclusion and exclusion criteria for smartphone app selection.**

**Inclusion criteria**

- Provides information about depression, including clinical presentation, diagnosis, and management (both pharmacological and nonpharmacological)
- Targets depression or includes depression information within a general mental health app
- Uploaded or updated from January 1, 2019, onward
- Downloaded at least 1000 times
- Available for free or requires payment to download, use, or expand functionalities (in-app purchases) and is available for download in the Apple App Store or Google Play
- Available in English

**Exclusion criteria**

- Presents an overview of common physical and mental disorders, using a glossary format
- Targets health care providers (eg, physicians, psychologists, and counselors) or the support network of a person with depression or consists exclusively of peer-support forums
- Offers teleconsultation services with physicians, psychologists, counselors, or other health care providers
- Consists of a stand-alone depression screening questionnaire, without education modules
- Offers complementary medicine, meditation, or lifestyle improvement
- Does not provide any depression-related information or includes non–health-related content (ie, music playlists, wallpapers, and so on)
- Requires an access code to log in, or could not be used after 2 log in attempts because of technical problems, or was withdrawn from the app store at the time of access

### App Assessment and Data Analysis

Apps were assessed by 2 assessors working in parallel using an iPhone 7 (iOS version 14.0.1) and a Sony XPERIA XZs (Android version 8.0.0) smartphone. If apps were available on both platforms, as per the 42matters search output, we assessed both versions and counted each version as an individual app. We used descriptive statistics to analyze the data. We compiled and tabulated the results and reported them as a narrative synthesis.

### Subgroup Analysis of Android Apps

We performed a subgroup analysis of Android apps to assess whether the number of app downloads was associated with the educational content or the quality or number of features offered by the apps. Apps were categorized into two groups: apps downloaded 1000-10,000 times and apps downloaded >10,000 times. The assessment included selected items from all 3 sections of the assessment. The data were tabulated and compared using a significance test for categorical variables: the chi-square test was used if each category contained >10 variables and a 2-tailed Fisher exact test was used if any of the categories in the contingency table contained <10 variables. Statistical significance was set at *P*<.05. Statistical analyses were performed in RStudio (R version 4.0.3). iOS apps were not included in this analysis because the Apple App Store does not include this information.

## Results

The app search retrieved 2218 results after removing duplicates and excluding apps with <1000 downloads, of which 245 were downloaded and 58 were included in the analysis. Figure 1 describes the app selection process.

### General Characteristics of Apps

Table 1 presents a summary of the characteristics of the included apps. Of the 58 apps included in this analysis, 29 (50%) were Android apps and 29 (50%) were iOS apps; 21% (12/58) of the apps were available on both platforms, and 60% (35/58) of the apps belonged to the health and fitness app store category. Of the 29 Android apps, 3 (10%) had been downloaded >1 million times [56-58]. Of the 58 included apps, 15 (26%) offered only education and information modules, whereas 37 (64%) offered education modules along with other mental health or depression management features. A simple interface that did not allow for user feedback or customization was offered by 41% (12/29) of the Android apps and 31% (9/29) of the iOS apps. More than 10% of the apps offered education modules targeted to specific user groups.

**Table 1:** General characteristics of apps (N=58).

Feature			Android (n=29), n (%)		iOS (n=29), n (%)		Total (N=58), n (%)
**App store category**							
	Education	4 (14)		0 (0)		4 (7)	
	Health and fitness	17 (59)		18 (62)		35 (60)	
	Lifestyle	1 (3)		1 (3)		2 (3)	
	Medical	7 (24)		10 (34)		17 (29)	
**App store rating, stars**							
	3.6 to 5	22 (76)		18 (62)		40 (69)	
	1 to 3.5	3 (10)		4 (14)		7 (12)	
	No ratings	4 (14)		7 (24)		11 (19)	
**App cost**							
	Free	18 (62)		15 (52)		33 (57)	
	Free + in-app purchases	11 (38)		14 (48)		25 (43)	
	Paid	0 (0)		0 (0)		0 (0)	
**Language**							
	English	26 (90)		23 (79)		49 (84)	
	English and other languages	3 (10)		6 (21)		9 (16)	
**Target user of the app**							
	No target user	25 (86)		26 (90)		51 (88)	
	Police officers	1 (3)		0 (0)		1 (2)	
	Veterans	1 (3)		1 (3)		2 (3)	
	Youth aged 12-18 years^a^	2 (7)		2 (7)		4 (7)	
**Scope of the app**							
	General mental health	21 (72)		18 (62)		39 (67)	
	Depression	8 (28)		11 (38)		19 (33)	
**Type of app**							
	Information and education	10 (34)		5 (17)		15 (26)	
	Disease management with education section	16 (55)		21 (72)		37 (64)	
	Education with disease management section	1 (3)		2 (7)		3 (5)	
	Multimedia education	2 (7)		1 (3)		3 (5)	
**Number of education topics**							
	<10	7 (24)		6 (21)		13 (22)	
	10-20	18 (62)		20 (69)		38 (66)	
	>20	4 (14)		3 (10)		7 (12)	
Emergency contact information for users at risk of suicide			16 (55)		19 (66)		35 (60)
Peer-support communities			2 (7)		2 (7)		4 (7)
Non–evidence-based information			10 (34)		2 (7)		12 (21)

^a^Youth was defined by the cutoff age provided by the apps.

### Depression Education Modules

#### Overview

Multimedia Appendix 2 presents a detailed description of the educational content in included apps. The apps offered a variety of information or educational topics, as summarized in Table 2. Most of the apps (51/58, 88%) included up to 20 educational topics. One in five apps provided non–evidence-based information, mostly in the form of personal opinions of the developers or columnists. Most of the apps informed users about the symptoms of depression and listed available treatments. Personal narratives on depression [51] were included only in approximately 20% of the apps.

**Table 2:** Depression education topics included in the apps (N=58).

Education topics included in the app				Android (n=29), n (%)		iOS (n=29), n (%)		Total (N=58), n (%)
**General information on depression**								
	Personal narratives of depression			6 (21)		6 (21)		12 (21)
	Depression is different from sadness			20 (69)		19 (66)		39 (67)
	Demographic and epidemiological facts			15 (52)		18 (62)		33 (57)
	Natural history of the disease			6 (21)		12 (41)		18 (31)
	Lists symptoms of depression			26 (90)		26 (90)		52 (90)
	Explains what recurrence and relapse are			9 (31)		5 (17)		14 (24)
	Addresses stigma linked to depression			11 (38)		11 (38)		22 (38)
	Mentions suicide risk linked to depression			23 (79)		20 (69)		43 (74)
**Screening for depression**								
	Describes diagnostic criteria of depression			7 (24)		6 (21)		13 (22)
	Provides reference to DSM-5^a^ or ICD-10^b^			3 (10)		3 (10)		6 (10)
	**Administers a screening questionnaire**			11 (38)		16 (55)		27 (47)
		PHQ-9^c^	5 (45)		8 (50)		13 (48)	
		PHQ-9 + GAD-7^d^	1 (9)		0 (0)		1 (4)	
		PHQ-9 + other validated questionnaires	1 (9)		2 (13)		3 (11)	
		Other validated questionnaires	1 (9)		2 (13)		3 (11)	
		Nonvalidated questionnaires	3 (27)		4 (25)		7 (26)	
	Explains the need for a confirmatory diagnosis after screening			7 (24)		3 (10)		10 (17)
**Treatment of depression**								
	Importance of seeking help			23 (79)		21 (72)		44 (76)
	Addresses phases and types of treatment (stepped or integrated treatment)			1 (3)		1 (3)		2 (3)
	Advises to seek specialist treatment			22 (76)		21 (72)		43 (74)
	Importance of involving support network			16 (55)		13(45)		29 (50)
	Importance of complying with treatment			8 (28)		8 (28)		16 (28)
	Lists available treatments			27 (93)		26 (90)		53 (91)
	**Treatments mentioned by the app**							
		Psychotherapy	3 (10)		4 (14)		7 (12)	
		Psychotherapy + pharmacotherapy	13 (45)		10 (34)		23 (40)	
		Psychotherapy + pharmacotherapy + others	7 (24)		7 (24)		14 (24)	
		Psychotherapy + others	0 (0)		2 (7)		2 (3)	
		Complementary medicine	3 (10)		1 (3)		4 (7)	
		Others	1 (3)		2 (7)		3 (5)	
	Importance of lifestyle changes			18 (62)		21 (72)		39 (67)
	Effects of physical exercise			19 (66)		20 (69)		39 (67)
	Offers emergency helpline phone numbers			16 (55)		19 (66)		35 (60)
	Prognosis in patients receiving treatment			7 (24)		13 (45)		20 (34)
	Addresses recovery and provides a hopeful outlook			3 (10)		3 (10)		6 (10)

^a^DSM-5: Diagnostic and Statistical Manual of Mental Disorders, Fifth Edition.

^b^ICD-10: International Classification of Diseases, Tenth Revision.

^c^PHQ-9: Patient Health Questionnaire, 9-item version.

^d^GAD-7: Generalized Anxiety Disorder, 7-item scale.

#### Symptoms and Natural History of Depression

Of the 58 included apps, 52 (90%) listed the symptoms of depression, whereas 39 (67%) stated the difference between depression and occasional low moods or sadness. Brief information on the epidemiology of depression was included in half of the assessed apps. Stigma associated with depression was mentioned by 38% (22/58) of the apps, whereas only 10% (6/58) included information on recovery.

Suicide risk associated with depression was mentioned by 74% (43/58) of the apps, generally as an item in a list of symptoms. Suicide prevention resources were offered by 60% (35/58) of the apps, including 55% (16/29) of the Android apps and 66% (19/29) of the iOS apps. The most common resource offered by the apps was information on crisis helplines (telephone numbers or website addresses).

#### Screening for Depression

Of the 58 included apps, 13 (22%) offered information on the diagnostic criteria for depression, 6 (10%) referenced psychiatric diagnostic manuals, and 10 (17%) explained the need to confirm diagnosis of depression after a positive screening test. Of the 58 apps, 27 (47%) apps administered a screening questionnaire for depression, commonly the Patient Health Questionnaire-9 [59]. Only 48% (13/27) of the apps responded to positive screening test scores by either offering self-management exercises for the user to engage with or suggesting consulting with a health care provider, commonly a physician, whereas the rest of the apps did not provide any feedback.

#### Treatment of Depression

Of the 58 included apps, 44 (76%) highlighted the importance of seeking help when affected by depression and consulting specialized providers if the symptoms were severe or persistent. Half of the Android apps and one-third of the iOS apps also emphasized the importance of involving members of the user’s support network. Few apps included providers’ contact information, and when such information was present, it was limited to contact information of the app development team; none of the apps included geolocated lists of health care providers by; for example, facilitating a search through navigation providers such as Google Maps.

More than 90% of the apps listed existing treatments for depression, without providing a description of what each category entailed. Psychotherapy was the most commonly mentioned treatment category (44/58, 76%), followed by medications (37/58, 64%), whereas 12% (7/58) of the apps included brain stimulation treatments as well. Nevertheless, few apps offered more comprehensive information on the different treatment modalities, including explanations of what each treatment involves and relevant examples (eg, types of psychotherapy and antidepressant groups). Information on psychotherapy modalities was offered by 34% (10/29) of the Android apps and 52% (15/29) of the iOS apps, whereas few Android or iOS apps offered information on medication types (8/58, 14%), side effects (13/58, 22%), nonaddictive nature of frequently used antidepressants (5/58, 9%), or gradual initiation and discontinuation of therapy (10/58, 17%).

Benefits of lifestyle modifications, including regular exercise, was mentioned by 67% (39/58) of the assessed apps, whereas complementary medicine, including St John’s wort, light therapy, and acupuncture, was mentioned by 7% (4/58) of the apps.

### Technical Aspects and Quality Assurance of the App

All assessed apps worked as intended and were generally easy to use. Data entry from users was not requested by 31% (9/29) of the iOS apps and 41% (12/29) of the Android apps (Table 3).

Of the 58 included apps, 13 (22%) available on both platforms did not include a privacy policy (6/29, 21%, Android apps and 7/29, 24%, iOS apps). These apps offered passive information that did not require data entry by users, except for 3% (1/29) of the Android apps and 7% (2/29) of the iOS apps, which requested the user’s email address to set up a password.

Of the 58 apps, 30 (52%) included references or the author’s signature or shared links to reputable websites for the information provided. Half of the Android apps and a quarter of the iOS apps did not declare developers’ affiliations, and 41% (24/58) of the apps included a disclaimer that the information provided did not replace a health care provider’s advice.

Of the 58 apps, 10 (17%) contained advertisements. These apps were free to download and use, except for an Android app that offered in-app purchases. The advertisements included in 75% (6/8) of the Android apps filled the screen and disrupted the use of the app, whereas 25% (2/8) of the apps included banner advertisements that allowed users to continue using the app. All advertisements were generally unrelated to the app content, promoting other Google Play apps, video games, beauty products, education support centers, and health care services such as dental services. Of these 8 Android apps, 1 (13%) promoted psychological services. In contrast, 100% (2/2) of the iOS apps offered banner advertisements that allowed for continued use of the app. The advertisements were not related to the app content.

**Table 3:** Technical features and quality assurance of included apps (N=58).

Characteristics				Android (n=29), n (%)		iOS (n=29), n (%)		Total (N=58), n (%)
**App credibility**								
	App content referenced or signed by the author			17 (59)		13 (45)		30 (52)
	Includes disclaimer: information does not replace health care provider’s advice			11 (38)		13 (45)		24 (41)
	**App development team included the following**							
		Government agency or academic institution or NGO^a^	4 (14)		8 (28)		12 (21)	
		Health care professional	11 (38)		14 (48)		25 (43)	
		Not declared	14 (48)		7 (24)		21 (36)	
**Data privacy**								
	Authentication required to access app			13 (45)		21 (72)		34 (59)
	**App includes a privacy policy**			23 (79)		22 (76)		45 (78)
		Presented before account creation	14 (61)		10 (45)		24 (53)	
		Explains how data are collected	23 (100)		22 (100)		45 (100)	
		Shares information with third party providers	20 (87)		20 (91)		40 (89)	
		Contact details of data protection officer provided	4 (17)		9 (41)		13 (29)	
	App allows users to share data			6 (21)		7 (24)		13 (22)
In-app advertisements (for details, please see text in this section)				8 (28)		2 (7)		10 (17)

^a^NGO: nongovernmental organization.

### Subgroup Analysis of Android Apps

Our subgroup analysis assessing the association between the Android apps’ popularity (as indexed by the number of app downloads) and the breadth and depth of the educational topics covered showed no statistically significant difference between apps downloaded <10,000 times and apps downloaded >10,000 times. Nonetheless, apps downloaded 1000-10,000 times provided non–evidence-based information more often than apps downloaded >10,000 times (7/15, 47% vs 3/14, 21%, respectively); however, this difference was not statistically significant. Of the 7 apps providing non–evidence-based information and downloaded <10,000 times, 2 (29%) belonged to the medical category in the app store. Of these 2 apps, 1 (50%) claimed that adhering to the *Law of Attraction* improve depression outcomes. Table 4 summarizes the analysis.

**Table 4:** Android apps subgroup analysis according to number of downloads (N=29).

Feature					>10,000 downloads (n=14), n (%)			1000-10,000 downloads (n=15), n (%)			*P* value^a^
**App store category**											.75
	Education			1 (7)			3 (20)				
	Health and fitness			9 (64)			8 (53)				
	Lifestyle			0 (0)			1 (7)				
	Medical			4 (29)			3 (20)				
**App store rating (stars)**											.09
	3.6 to 5			13 (93)			9 (60)				
	1 to 3.5			1 (7)			2 (13)				
	No ratings			0 (0)			4 (27)				
**App cost**											.06
	Free			6 (43)			12 (80)				
	Free + in-app purchase			8 (57)			3 (20)				
	Paid			0 (0)			0 (0)				
**Type of app**											.07
	Information and education			2 (14)			8 (53)				
	Disease management with education section			10 (71)			6 (40)				
	Education disease management section			1 (7)			0 (0)				
	Multimedia education			1 (7)			1 (7)				
**Number of education topics**											.66
	<10			3 (21)			4 (27)				
	10-20			8 (57)			10 (67)				
	>20			3 (21)			1 (7)				
Peer-support communities					2 (14)			0 (0)			.22
Non–evidence-based information					3 (21)			7 (47)			.25
**Depression education**											
	**General information on depression**										
		Personal narratives of depression	4 (29)			2 (13)			.39		
		Depression is different from sadness	11 (79)			9 (60)			.43		
		Demographic and epidemiological facts	9 (64)			6 (40)			.35		
		Natural history of the disease	6 (43)			0 (0)			*.006*		
		Lists symptoms of depression	14 (100)			12 (80)			.22		
		Explains what recurrence and relapse are	4 (29)			5 (33)			.99		
		Addresses stigma linked to depression	7 (50)			4 (27)			.36		
		Mentions suicide risk linked to depression	12 (86)			11 (73)			.65		
	**Screening of depression**										
		Describes diagnostic criteria of depression	4 (29)			3 (20)			.69		
		Administers a screening questionnaire	7 (50)			4 (27)			.36		
		Explains need for a confirmatory diagnosis after screening	4 (29)			3 (20)			.68		
	**Treatment of depression**										
		Importance of seeking help	12 (86)			11 (73)			.65		
		Advises to seek specialist treatment	9 (64)			13 (87)			.21		
		Importance of involving support network	9 (64)			7 (47)			.56		
		Importance of complying with treatment	4 (29)			4 (27)			.99		
		Lists available treatments	11 (79)			12 (80)			.99		
		Importance of lifestyle changes	10 (71)			8 (53)			.53		
		Lists complementary medicine options	0 (0)			3 (20)			.22		
		Offers emergency helpline phone numbers	10 (71)			6 (40)			.18		
		Prognosis in patients receiving treatment	5 (36)			0 (0)			*.02*		
		Addresses recovery and provides a hopeful outlook	2 (14)			2 (13)			.99		
**App credibility**											
	App content referenced or signed by author			12 (86)			5 (33)			*.012*	
	Include disclaimer: information does not replace health care provider’s advice			7 (50)			4 (27)			.36	
	**App development team included the following**										.11
		Government agency or academic institution or NGO^b^	2 (14)			2 (13)					
		Health care professional	8 (57)			3 (20)					
		Not declared	4 (29)			10 (67)					
**Data privacy**											
	Authentication required to access app			9 (64)			4 (27)			.10	
	**App includes a privacy policy**			12 (86)			11 (73)			.65	
		Presented before account creation	9 (64)			5 (33)			.20		
		Explains how data are collected	12 (86)			11 (73)			.65		
		Shares information with third party providers	10 (71)			10 (67)			.99		
		Contact details of data protection officer provided	4 (29)			0 (0)			*.04*		
In-app advertisements					2 (14)			6 (40)			.21

^a^Statistically significant values in italics.

^b^NGO: nongovernmental organization.

## Discussion

### Principal Findings

To our knowledge, this is the first systematic assessment of the information on depression in existing mental health and depression apps. Our findings suggest that the information included in these apps is often limited and not aligned with evidence. Most of the assessed apps offered brief, factual information on symptoms and treatment options, with only a few apps addressing all aspects of disease presentation and management or including information on recovery or providing personal narratives of people living with depression. The inclusion of personal accounts of people living with, and recovering from, a mental health disorder has been associated with increased understanding of the mental health disorder and recovery process, validation of the personal experience, and reduction of stigma [28,60].

Patient education is one of the pillars of effective depression management resulting in better treatment compliance and improved outcomes [9,11], particularly if they are complemented with personal narratives and positive messages emphasizing recovery [51]. Adequate health literacy is associated with tolerance and acceptance of people living with a mental disorder [16], increased help-seeking behavior [16], and adherence to treatment [61,62], and it is a prerequisite to engage in shared-decision models of care [63]. Yet, 25% of the potentially eligible apps, including some of the most popular mental health apps with >1 million downloads each, were excluded in the second round of screening because they did not provide any information on depression.

Access to mental health care is often inadequate, particularly in low- and middle-income countries [3] and among people of lower socioeconomic status as well as those living in remote areas in high-income countries [64,65].

Mental health apps may play an important role in improving access to mental health care, supported by an increasing expansion of mobile network coverage to remote areas worldwide [31]. However, our assessment shows that apps currently available in public app marketplaces do not offer a holistic, evidence-based self-management program to meet these health needs. For example, less than half of the assessed apps include a screening test for depression and only a subset of these apps offer follow-up recommendations to users potentially living with depression. Of the 58 assessed apps, 12 (21%) offered non–evidence-based information, including developers’ or columnists’ personal views on depression, and suggested scientifically unproved treatments. Of these 12 apps, 2 (17%) were classified as *medical* in the app store. Lack of evidence-based information in health apps is a recurrent theme in most systematic assessments performed by our group [45,46,48] and others [42,66,67], revealing that current app development and publication processes overseen by app stores might not be suitable for health apps. We previously highlighted the lack of governance and quality assurance of the health app industry [48], which we believe constrains the further development of health apps as genuine tools to support access to mental health care. Therefore, it is imperative to develop a multifaceted approach involving the research community, commercial app developers, app store managers, and official regulatory bodies to define development and publication regulatory frameworks for health apps.

Our subgroup assessment of Android apps suggests an association between the number of downloads and the overall quality of the apps. In the analysis, apps downloaded >10,000 times were more trustworthy, included more evidence-based content, presented better compliance with data privacy and security, presented references to endorse claims, and incorporated health care providers into the app development team more often than apps downloaded <10,000 times. Excluding app credibility, the differences were not statistically significant, probably because of the small size of the sample.

In this study, we used an established systematic assessment approach, which is based on the rigorous systematic review and which we have applied to various health domains [46-48] over the years. This methodology included the use of a commercial database to search for eligible apps, which provides a wider geographical scope for the search, and the development of exhaustive assessment criteria using renowned evidence-based clinical guidelines and our center’s criteria for technical and quality assurance of apps.

There are limitations to our research as well. The search strategy included depression-related terms and omitted education-based terminology; thus, we may have omitted some relevant apps. However, the inclusion of such terms would have greatly increased the overall number of retrieved apps and would have also led to retrieval of many irrelevant apps because the database used for app retrieval does not allow the use of search strings; instead, it retrieves individual results for each search term. We restricted our search to apps for mental health, excluding apps offering medical or general health advice that may have presented summaries on depression presentation or management. Our assessment was limited to apps downloaded at least 1000 times, which may have omitted newly launched apps that have yet to reach the minimum required number of downloads. We also restricted our search to apps in English, potentially excluding relevant apps in languages other than English.

### Conclusions

Information in mental health and depression apps is often brief and incomplete, with 1 in 5 apps providing non–evidence-based information. Given the unmet needs and stigma associated with depression, it is imperative that apps seize the opportunity to offer quality, evidence-based education and point users to relevant resources. A multistakeholder consensus on a more stringent development and publication process for mental health apps is essential.

## Appendix

Multimedia Appendix 1 Depression education topics assessment criteria.

Multimedia Appendix 2 Characteristics of included apps.
