# Supplementary material for: Education on Depression in Mental Health Apps: Systematic Assessment of Characteristics and Adherence to Evidence-Based Guidelines
Source: J Med Internet Res. 2022 Mar 9;24(3):e28942. doi: 10.2196/28942 (PMC8943550; doi:10.2196/28942)
Supplement: Multimedia Appendix 1 [file jmir_v24i3e28942_app1.docx]

**Multimedia Appendix 1**: Depression education topics assessment criteria

| Field | Ref | Description | Options | Justification/Evidence |
| --- | --- | --- | --- | --- |
| 2-1 Symptoms and natural history of depression | 2-1-1 | Does the app clarifies that "sadness" is not the same as "depression"? | Yes/No | "Educating the patient about the illness helps clarify uncertainty and misconceptions. Depression should be explained as a medical illness that is associated with changes in neurochemicals and brain functioning" [1,2] |
|  | 2-1-2 | Does the app reports epidemiological data on depression (e.g. depression is very common, affects more women than men, onset in early adulthood, etc.) | Yes/No | "Depression is the most common mental disorder in community settings and is a major cause of disability across the world…" [1-4] |
|  | 2-1-3 | Does the app list the symptoms of depression? | Yes/No | "Education about the symptoms and treatment of major depressive disorder should be provided in language that is readily understandable to the patient... education about MDD should address the need for a full acute course of treatment, the risk of relapse, the early recognition of recurrent symptoms, and the need to seek treatment as early as possible to reduce the risk" [3]  "Educating the patient about the illness helps clarify uncertainty and misconceptions. Depression should be explained as a medical illness that is associated with changes in neurochemicals and brain functioning." [1,2,4] |
|  | 2-1-4 | Does the app inform users of the natural history of the disease without treatment? | Yes/No |  |
|  | 2-1-5 | Does the app explain the prognosis of the disease in untreated patients? | Yes/No |  |
|  | 2-1-6 | Does the app explain the prognosis of the disease in patients receiving treatment? | Yes/No |  |
|  | 2-1-7 | Does the app explains what recovery is? | Yes/No | “Once the patient has been asymptomatic for at least 6 months … **recovery** from the episode is declared.” [3]  “The end goal is achieving recovery to premorbid level of functioning” [4]  “…explore treatment options … that recovery is possible” [2] |
|  | 2-1-8 | Does the app explains that the end goal of treatment is to achieve recovery? | Yes/No |  |
|  | 2-1-9 | Does the app explains what a relapse is? | Yes/No | "...education about MDD should address … the risk of relapse…" "Before the discontinuation of active treatment, patients should be informed of the potential for a depressive relapse." [1-4] |
|  | 2-1-10 | Does the app explains what recurrence is? | Yes/No | "...education about MDD should address … the early recognition of recurrent symptoms…" "Major depressive disorder is … recurrent in 35%." "After three episodes, the risk of recurrence approaches 100% in the absence of prophylactic treatment." [1-4] |
|  | 2-1-11 | Does the app explain what comorbid conditions are? | Yes/No | "… and the presence of co-occurring psychiatric disorders. … Co-occurring general medical conditions are common and can influence the diagnosis of major depressive disorder as well as choices of treatment. … Major depressive disorder is also associated with significant medical comorbidity and complicates recovery from other medical illnesses," [3] |

**Multimedia Appendix 1**: Depression education topics assessment criteria (continuation)

| Field | Ref | Description | Options | Justification/Evidence |
| --- | --- | --- | --- | --- |
|  | 2-1-12 | Does the app explain risk of suicide? | Yes/No | "The most serious complication of a major depressive episode is suicide" [1,3] |
|  | 2-1-13 | Does the app address stigma in depression and mental health disorders in general? | Yes/No | "The assessment and treatment of major depressive disorder should consider … degree to which psychiatric illness is stigmatized" [3]  "The stigma associated with depression cannot be ignored…" "When working with people with depression and their families or carers ... be aware that stigma and discrimination can be associated with a diagnosis of depression" [2] |
|  | 2-1-14 | Does the app address the importance of seeking help for people suffering from depression? | Yes/No | The stigma associated with mental health problems … may partly account for the reluctance of people with depression to seek help "People may have beliefs that prevent them from seeking help for depression…" [2] |
|  | 2-1-15 | Does the app includes personal stories of people recovering from depression? | Yes/No | “…interventions with social contact or first person narratives were more effective than others…” [5] |
| 2-2 Screening of depression | 2-2-1 | Does the app inform users of the diagnostic criteria of depression? | Yes/No | [3,4] use the DSM criteria (DSM-IV in [3] and DSM-5 in [4])  [1,2] mention DSM-IV and ICD-10 |
|  | 2-2-2 | Are DSM-5 or ICD-10 referenced as the source of the diagnostic criteria listed by the app? | Yes/No | "… psychiatrist has diagnosed major depressive disorder, according to the criteria defined in DSM-IV-TR, in an adult patient…" [3] DSM-5 referenced in [4] |
|  | 2-2-3 | Does the app explain what self-reported screening questionnaires are? | Yes/No | "A range of self-rated and observer-rated questionnaires are available for the evaluation of depression…" [1]  "If a person answers 'yes' to either of the depression identification questions (see 1.3.1.1), a practitioner who is competent to perform a mental health assessment should review the person's mental state..." "When assessing a person with suspected depression, consider using a validated measure... to inform and evaluate treatment..." [2] |
|  | 2-2-4 | Does the app use any particular self-reported questionnaire? | Yes/No |  |
|  | 2-2-5 | If yes, could you please specify which questionnaire/s the app use? | Free text |  |
|  | 2-2-6 | If the questionnaire suggests the user may be depressed, does the app suggest to consult with a HCP? | Yes/No |  |
|  | 2-2-7 | Does the app explain the need of confirmatory diagnosis after positive screening? | Yes/No |  |
|  | 2-2-8 | Does the app emphasises that on certain occasions it is very difficult to reach a definitive diagnosis? | Yes/No | "… it is sometimes difficult to make a definitive diagnosis of a mood disorder." [4] |
| 2-3 Treatment of depression | 2-3-1 | Does the app explains users the different phases of depression treatment (acute, continuation and maintenance)? | Yes/No | "Treatment in the acute phase should be aimed at inducing remission ... and achieving a full return to the patient’s baseline level of functioning" "During the continuation phase of treatment, the patient should be carefully monitored for signs of possible relapse" "... proceed to the maintenance phase of treatment after completing the continuation phase" [1-4] |

**Multimedia Appendix 1**: Depression education topics assessment criteria (continuation)

| Field | Ref | Description | Options | Justification/Evidence |
| --- | --- | --- | --- | --- |
|  | 2-3-2 | Does the app explain what “stepped treatment” means? | Yes/No | "In stepped care the least intrusive, most effective intervention is provided first; if a person does not benefit from the intervention initially offered, or declines an intervention, they should be offered an appropriate intervention from the next step..." [2] "...step-wise management of major depressive disorder…" [4] |
|  | 2-3-3 | Does the app explains what is an "integrated care team" and who its members are? | Yes/No | "The optimal management of people with ... mood disorders requires ... the involvement of several health care professionals including a general practitioner (GP), mental health nurse, psychiatrist and psychological counsellors but the active partnership of family, carers and support groups as part of an integrated care team" [4] |
|  | 2-3-4 | Does the app advise the user of the benefits of involving family or other members of his/her support network in the treatment? | Yes/No | "Where indicated and with patients’ agreement, involve family members or friends in the care of people with depression so that there is adequate support. [1,3] |
|  | 2-3-5 | Does the app emphasizes the need to adhere and complete the course of treatment as advised by the treating healthcare provider? | Yes/No | "… education about MDD should address the need for a full acute course of treatment,…" [1-4] |
|  | 2-3-6 | Does the app explains that establishing a good therapeutic alliance improves the treatment outcomes? | Yes/No | "The alliance itself may be the primary active therapeutic agent even for patients who receive monotherapy with medication" [1-4] |
|  | 2-3-7 | Does the app list the treatment options available for depressed patients? | Yes/No | "Acute phase treatment may include pharmacotherapy, depression-focused psychotherapy, the combination of medications and psychotherapy, or other somatic therapies such as electroconvulsive therapy (ECT), transcranial magnetic stimulation (TMS), or light therapy,..." [1,3,4]  "… In patients who prefer complementary and alternative therapies, S-adenosyl methionine (SAMe) [III] or St. John’s wort [III] might be considered…" "ECT is recommended ... for patients with severe major depressive disorder that is not responsive to psychotherapeutic and/or pharmacological interventions..." [1] "Bright light therapy might be used to treat seasonal affective disorder as well as non-seasonal depression..." [3,4] |
|  | 2-3-8 | What types of interventions are mentioned in the app? | Psychological interventions/ Pharmacological interventions/ ECT/ TMS/ Complementary medicine/ Light therapy/ Others |  |
|  | 2-3-9 | What psychological interventions are mentioned in the app? | Free text (e.g. CBT, BA, IPT, others) | "… with clinical evidence supporting the use of cognitive-behavioral therapy (CBT) [I], interpersonal psychotherapy [I], psychodynamic therapy [II], and problem-solving therapy [III] in individual [I] and in group [III] formats…" [1-4] |
|  | 2-3-10 | Does the app explains the characteristics of each psychological intervention? | Yes/No |  |

**Multimedia Appendix 1**: Depression education topics assessment criteria (continuation)

| Field | Ref | Description | Options | Justification/Evidence |
| --- | --- | --- | --- | --- |
|  | 2-3-11 | What pharmacological interventions are mentioned in the app? | Free text (e.g. SSRIs, TCAs, others) | "... a selective serotonin reuptake inhibitor (SSRI), serotonin norepinephrine reuptake inhibitor (SNRI), mirtazapine, or bupropion is optimal [I]. ... the use of nonselective monoamine oxidase inhibitors (MAOIs) (e.g., phenelzine, tranylcypromine, isocarboxazid) should be restricted.." [1-4] |
|  | 2-3-12 | Does the app explain the characteristics of each medication group? | Yes/No |  |
|  | 2-3-13 | Does the app explain the side effects of each medication group? | Yes/No |  |
|  | 2-3-14 | Does the app explain that psychoactive medications (e.g. depression-specific medications) should be started and withdrawn gradually? | Yes/No | "Patients should also be told about the need to taper antidepressants, rather than discontinuing them precipitously, to minimize the risk of withdrawal symptoms or symptom recurrence" [3] "...cessation of medication should occur slowly with a taper over an extended period of time because withdrawal symptoms may be experienced." [4] "When stopping an antidepressant, gradually reduce the dose, normally over a 4-week period..." [2] |
|  | 2-3-15 | Does the app clarify that current evidence consider antidepressants non-addictive? | Yes/No | "Common misperceptions about antidepressants (e.g., they are addictive) should be clarified" [1,3] "… antidepressants are not associated with addiction." [2] |
|  | 2-3-16 | Does the app mention lifestyle changes as an important treatment adjuvant for depression? | Yes/No | "Patient education also includes general promotion of healthy behaviors such as exercise, good sleep hygiene, good nutrition, and decreased use of tobacco, alcohol, and other potentially deleterious substances" [1,3,4] |
|  | 2-3-17 | Does the app explains the importance of exercise and an active lifestyle in the treatment of depression? | Yes/No | "Data generally support at least a modest improvement in mood symptoms for patients with major depressive disorder who engage in aerobic exercise or resistance training. Regular exercise may also reduce the prevalence of depressive symptoms in the general population." "If a patient with mild depression wishes to try exercise alone for several weeks as a first intervention, there is little to argue against it…" [1-4] |
|  | 2-3-18 | Does the app mentions complementary medicine as a treatment option for depression? | Yes/No | "Some of these modalities can be recommended with enthusiasm for their general health benefits; however, patients should be informed that evidence for their antidepressant efficacy as monotherapy is limited or absent." "Bright light therapy might be used to treat seasonal affective disorder as well as non-seasonal depression." [3,4] |
|  | 2-3-19 | If yes, could you please specify which complementary medicine modalities are explained in the app? | Free text |  |
|  | 2-3-20 | Does the app explains the need to continuously monitor and re-evaluate treatment response? | Yes/No | "Continued monitoring of co-occurring psychiatric and/or medical conditions is also essential to developing and refining a treatment plan for an individual patient" [3,4] |

**Multimedia Appendix 1**: Depression education topics assessment criteria (continuation)

| Field | Ref | Description | Options | Justification/Evidence |
| --- | --- | --- | --- | --- |
|  | 2-3-21 | Does the app advice the user when to seek specialized care? | Yes/No | "...where there is diagnostic complexity, a high risk of self-harm, non-response or severe debility, referral for psychiatric assessment is indicated…" [4] "A referral to a specialist is indicated when ... associated with high suicide risk, in severe postnatal depression... psychotic symptoms present and ... symptoms suggestive of bipolar disorder. [1] "Referral to specialist mental health services should normally be for people with depression who are at significant risk of self-harm, have psychotic symptoms, require complex multi-professional care, or where an expert opinion on treatment and management is needed." [2] |
|  | 2-3-22 | Does the app offer emergency resources for users at risk of suicide? | Yes/No | “always ask people with depression directly about suicidal ideation and intent. If there is a risk of self-harm or suicide:  assess whether the person has adequate social support and is aware of sources of help” [2] |
|  | 2-3-23 | If yes, please list the resources offered by the app | Free text |  |

HCP: healthcare provider, ECT: Electroconvulsive therapy, TMS: Transcranial Magnetic Stimulation, CBT: Cognitive Behavioral Therapy, BA: Behavioral activation, IPT: Interpersonal psychotherapy, SSRI: Selective serotonin reuptake inhibitors, TCA: Tricyclic antidepressants

References

1. Ministry of Health Singapore. Depression - MOH Clinical Practice Guidelines 1/2012. In: Ministry of Health, ed. Singapore2012.
2. NICE. Depression in adults: recognition and management. In. London: National Institute for Health and Care Excellence; 2009.
3. American Psychiatric Association. Practice guideline for the treatment of patients with major depressive disorder*.* 3rd ed. ed. Arlington (VA): American Psychiatric Association (APA); 2010.
4. Malhi GS, Bassett D, Boyce P, et al. Royal Australian and New Zealand College of Psychiatrists clinical practice guidelines for mood disorders. Aust N Z J Psychiatry. 2015;49(12):1087-1206.
5. Thornicroft G, Mehta N, Clement S, et al. Evidence for effective interventions to reduce mental-health-related stigma and discrimination. Lancet. 2016.387(10023): 1123-1132.
