# Supplementary material for: Education on Depression in Mental Health Apps: Systematic Assessment of Characteristics and Adherence to Evidence-Based Guidelines
Source: J Med Internet Res. 2022 Mar 9;24(3):e28942. doi: 10.2196/28942 (PMC8943550; doi:10.2196/28942)
Supplement: Multimedia Appendix 2 [file jmir_v24i3e28942_app2.docx]

**Multimedia Appendix 2**: Characteristics of included apps

| **App characteristics** | | | | | | **Depression education modules content** | | | | | | | | | | |
| --- | --- | --- | --- | --- | --- | --- | --- | --- | --- | --- | --- | --- | --- | --- | --- | --- |
| **App name** | **Platform** | **# of downloads** | **Type of**  **app** | **Developer team includes:**  **Country** | **Contains evidence-based information** | **Sadness different from depression** | **List depression symptoms** | **Explain relapse/ recurrence** | **Address stigma** | **Diagnostic criteria** | **Administer screening tool** | **Explain treatment principles** | **List treatment options** | **Antidepress ants are non-addictive** | **Lifestyle changes to improve symptoms** | **Emergency help for suicidal users** |
| 7 Cups: Anxiety & Stress Chat | Android | 1,000,000+ | Disease management + education | HCP  USA | Yes | Yes | Yes | No | Yes | No | No | No | Yes | No | Yes | Yes |
| All Life Skills You Need | Android | 1,000+ | Information & Education | Nil  Unknown | Yes | Yes | Yes | Yes | Yes | Yes | No | No | Yes | Yes | Yes | Yes |
| Anxiety & Depression Symptoms | Android | 1,000+ | Information & Education | Nil  Canada | No | Yes | Yes | No | no | No | No | No | Yes | No | No | Yes |
| Ashley – Your Emotional Support | Android | 10,000+ | Disease management + education | HCP  USA | Yes | Yes | Yes | Yes | Yes | No | No | No | Yes | No | No | Yes |
| Backup Buddy [SSP] | Android | 1,000+ | Disease management + education | Gov Ag  UK | Yes | No | Yes | No | No | Yes | No | No | No | No | Yes | Yes |
| BeSerene \| Mind Training and Stress Management App | Android | 5,000+ | Disease management + education | HCP  India | Yes | No | Yes | No | No | No | Non-validated | No | Yes | No | Yes | No |
| CBT Tools for Healthy Living, Self-help Mood Diary | Android | 100,000+ | Disease management + education | HCP  USA | Yes | Yes | Yes | No | Yes | Yes | No | No | Yes | No | Yes | No |
| Clinical Depression | Android | 1,000+ | Information & Education | Nil  Unknown | Yes | Yes | Yes | No | No | No | No | No | Yes | Yes | Yes | No |
| Cognitive Behavioral Therapy - Knowledge and Tips | Android | 1,000+ | Information & Education | Nil  Unknown | No | No | No | Yes | No | No | No | No | Yes | No | Yes | No |
| Dealing with Depression | Android | 5,000+ | Information & Education | Nil  Unknown | No | Yes | Yes | Yes | No | No | No | No | Yes | No | Yes | No |
| Easy Psychiatry : Online Clinic, Affirmations | Android | 5,000+ | Disease management + education | Nil  Nigeria | No | Yes | Yes | No | No | No | No | No | Yes | No | No | No |
| Emotionally Intelligent | Android | 1,000+ | Information & Education | Nil  Romania | No | No | No | Yes | No | No | No | No | Yes | No | Yes | No |

**Multimedia Appendix 2**: Characteristics of included apps (continuation)

| **App characteristics** | | | | | | **Depression education modules content** | | | | | | | | | | |
| --- | --- | --- | --- | --- | --- | --- | --- | --- | --- | --- | --- | --- | --- | --- | --- | --- |
| **App name** | **Platform** | **# of downloads** | **Type of**  **app** | **Developer team includes:**  **Country** | **Contains evidence-based information** | **Sadness different from depression** | **List depression symptoms** | **Explain relapse/ recurrence** | **Address stigma** | **Diagnostic criteria** | **Administer screening tool** | **Explain treatment principles** | **List treatment options** | **Antidepress ants are non-addictive** | **Lifestyle changes to improve symptoms** | **Emergency help for suicidal users** |
| InnerHour Self-Care Therapy - Anxiety & Depression | Android | 500,000+ | Disease management + education | HCP  India | No | Yes | Yes | No | Yes | Yes | Non-validated | No | Yes | No | Yes | No |
| Jockey Club TourHeart Project | Android | 1,000+ | Disease management + education | Acad Inst  Hong Kong | Yes | Yes | Yes | No | Yes | Yes | PHQ-9, GAD-7, Flourishing Scale | No | Yes | No | No | Yes |
| Joyable: An AbleTo Program | Android | 1,000+ | Disease management + education | HCP  USA | Yes | Yes | Yes | No | No | No | PHQ-9 | No | No | No | No | Yes |
| LifeArmor | Android | 10,000+ | Education + disease management | Gov Ag  USA | Yes | Yes | Yes | No | Yes | No | PHQ-9 | No | No | No | No | Yes |
| Moodpath | Android | 1,000,000+ | Disease management + education | HCP  Germany | Yes | Yes | Yes | Yes | No | Yes | Non-validated | Yes | Yes | No | No | Yes |
| MoodTools - Depression Aid | Android | 100,000+ | Disease management + education | Nil  USA | Yes | Yes | Yes | Yes | No | No | PHQ-9 | No | Yes | No | Yes | Yes |
| Overcoming Depression | Android | 1,000+ | Information & Education | Nil  Unknown | No | No | No | No | No | No | No | No | No | No | No | No |
| Personality Disorders Guide | Android | 1,000+ | Multimedia education | Nil  Unknown | Yes | No | Yes | Yes | No | No | No | No | Yes | No | No | No |
| Psychiatry Pro-Diagnosis, Info, Treatment, CBT & DBT | Android | 10,000+ | Information & Education | Nil  Unknown | Yes | Yes | Yes | Yes | No | Yes | Non-validated | No | Yes | No | Yes | Yes |
| PUSH-D | Android | 1,000+ | Disease management + education | HCP  India | Yes | Yes | Yes | No | Yes | No | PHQ-9, GAD-7 | No | Yes | Yes | No | Yes |
| Reduce Depression | Android | 1,000+ | Information & Education | Nil  Unknown | No | Yes | Yes | No | Yes | No | No | No | Yes | No | Yes | No |
| Self Help | Android | 10,000+ | Information & Education | Gov Ag  UK | Yes | No | Yes | No | No | No | No | No | Yes | No | Yes | Yes |
| Sintelly – Emotional Intelligence & Psychology | Android | 500,000+ | Multimedia education | Nil  Croatia | No | Yes | Yes | No | No | No | No | No | Yes | No | Yes | No |

**Multimedia Appendix 2**: Characteristics of included apps (continuation)

| **App characteristics** | | | | | | | **Depression education modules content** | | | | | | | | | | | | | | | | | | | | |
| --- | --- | --- | --- | --- | --- | --- | --- | --- | --- | --- | --- | --- | --- | --- | --- | --- | --- | --- | --- | --- | --- | --- | --- | --- | --- | --- | --- |
| **App name** | **Platform** | **# of downloads** | **Type of**  **app** | **Developer team includes:**  **Country** | **Contains evidence-based information** | | **Sadness different from depression** | | **List depression symptoms** | | **Explain relapse/ recurrence** | | **Address stigma** | | **Diagnostic criteria** | | **Administer screening tool** | | **Explain treatment principles** | | **List treatment options** | | **Antidepress ants are non-addictive** | | **Lifestyle changes to improve symptoms** | | **Emergency help for suicidal users** |
| Switch | Android | 10,000+ | Disease management + education | HCP  USA | No | No | | Yes | | No | | Yes | | No | | No | | No | | No | | No | | Yes | | No | |
| WellTrack - Interactive Self-Help Therapy | Android | 10,000+ | Disease management + education | Nil  Canada | Yes | Yes | | Yes | | No | | No | | No | | DASS21 | | No | | Yes | | No | | No | | Yes | |
| What's Up? - A Mental Health App | Android | 500,000+ | Disease management + education | HCP  Australia | Yes | No | | Yes | | No | | No | | No | | No | | No | | No | | No | | Yes | | Yes | |
| Youper - Feel your best | Android | 1,000,000+ | Disease management + education | HCP  USA | Yes | Yes | | Yes | | No | | Yes | | No | | PHQ-9 | | No | | Yes | | No | | Yes | | Yes | |
| 7 Cups: Anxiety & Stress Chat | iOS | NA | Disease management + education | HCP  USA | Yes | Yes | | Yes | | No | | Yes | | No | | No | | No | | Yes | | No | | Yes | | Yes | |
| anxietyhelper | iOS | NA | Education + disease management | Nil  USA | Yes | No | | Yes | | No | | No | | No | | No | | No | | Yes | | No | | Yes | | Yes | |
| Bee Appy | iOS | NA | Multimedia education | NGO  Scotland | Yes | No | | Yes | | No | | No | | No | | No | | No | | Yes | | No | | Yes | | Yes | |
| BeSerene \| Mind Training App | iOS | NA | Disease management + education | HCP  India | Yes | No | | Yes | | No | | No | | No | | Non-validated | | No | | Yes | | No | | Yes | | No | |
| Bloom: CBT Therapy & Self-Care | iOS | NA | Disease management + education | Nil  USA | Yes | Yes | | No | | No | | Yes | | No | | No | | No | | Yes | | No | | Yes | | No | |
| Braive: Mental Health Help | iOS | NA | Disease management + education | HCP  Norway | Yes | Yes | | Yes | | No | | Yes | | No | | PHQ-9, DASS21 | | No | | Yes | | No | | No | | Yes | |
| Depression Aide Lite | iOS | NA | Disease management + education | Nil  Unknown | Yes | No | | No | | No | | No | | No | | PHQ-9 | | No | | Yes | | No | | Yes | | Yes | |
| Depression Manager | iOS | NA | Disease management + education | Nil  USA | Yes | Yes | | Yes | | Yes | | No | | Yes | | No | | No | | Yes | | Yes | | Yes | | Yes | |
| Electroconvulsive Therapy (ECT) | iOS | NA | Information & Education | Gov Ag  UK | Yes | No | | Yes | | No | | No | | No | | No | | No | | Yes | | No | | No | | No | |
| Flow – Depression | iOS | NA | Disease management + education | HCP  Sweden | Yes | Yes | | Yes | | No | | No | | Yes | | MADRS | | No | | Yes | | No | | Yes | | Yes | |

**Multimedia Appendix 2**: Characteristics of included apps (continuation)

| **App characteristics** | | | | | | | **Depression education modules content** | | | | | | | | | | | | | | | | | | | | |
| --- | --- | --- | --- | --- | --- | --- | --- | --- | --- | --- | --- | --- | --- | --- | --- | --- | --- | --- | --- | --- | --- | --- | --- | --- | --- | --- | --- |
| **App name** | **Platform** | **# of downloads** | **Type of**  **app** | **Developer team includes:**  **Country** | **Contains evidence-based information** | | **Sadness different from depression** | | **List depression symptoms** | | **Explain relapse/ recurrence** | | **Address stigma** | | **Diagnostic criteria** | | **Administer screening tool** | | **Explain treatment principles** | | **List treatment options** | | **Antidepress ants are non-addictive** | | **Lifestyle changes to improve symptoms** | | **Emergency help for suicidal users** |
| Happier You-Community, therapy | iOS | NA | Disease management + education | HCP  UK | Yes | No | | Yes | | Yes | | No | | No | | PHQ-9 | | No | | Yes | | No | | Yes | | No | |
| Happify: for Stress & Worry | iOS | NA | Disease management + education | HCP  USA | Yes | Yes | | Yes | | No | | Yes | | Yes | | Non-validated | | No | | Yes | | No | | Yes | | Yes | |
| InnerHour - Live Happier | iOS | NA | Disease management + education | HCP  India | No | Yes | | Yes | | No | | Yes | | Yes | | Non-validated | | No | | Yes | | No | | Yes | | No | |
| Jockey Club TourHeart Project | iOS | NA | Disease management + education | Acad Inst  Hong Kong | Yes | Yes | | Yes | | No | | Yes | | Yes | | PHQ-9, GAD-7, Flourishing Scale | | No | | Yes | | No | | No | | Yes | |
| Joyable: An AbleTo Program | iOS | NA | Disease management + education | HCP  USA | Yes | Yes | | Yes | | No | | No | | No | | PHQ-9 | | No | | No | | No | | No | | Yes | |
| LifeArmor | iOS | NA | Education + disease management | Gov Ag  USA | Yes | Yes | | Yes | | No | | Yes | | No | | PHQ-9 | | No | | No | | No | | No | | Yes | |
| Mersey Care Self Help | iOS | NA | Information & Education | Gov Ag  UK | Yes | No | | Yes | | No | | No | | No | | No | | No | | Yes | | Yes | | Yes | | No | |
| Mood Coach | iOS | NA | Disease management + education | Gov Ag  USA | Yes | Yes | | Yes | | No | | No | | No | | PHQ-9 | | No | | Yes | | No | | Yes | | Yes | |
| Moodpath - Depression & Anxiety Test | iOS | NA | Disease management + education | HCP  Germany | Yes | Yes | | Yes | | Yes | | No | | Yes | | Non-validated | | Yes | | Yes | | No | | No | | Yes | |
| MoodTools - Depression Aid | iOS | NA | Disease management + education | Nil  USA | Yes | Yes | | Yes | | Yes | | No | | No | | PHQ-9 | | No | | Yes | | No | | Yes | | Yes | |
| MSP Self Help | iOS | NA | Information & Education | Gov Ag  UK | Yes | No | | Yes | | No | | No | | No | | No | | No | | Yes | | Yes | | Yes | | No | |
| PsychScope | iOS | NA | Disease management + education | HCP  Canada | Yes | Yes | | Yes | | Yes | | Yes | | No | | No | | No | | Yes | | No | | No | | No | |
| Self Help | iOS | NA | Information & Education | Gov Ag  UK | Yes | No | | Yes | | No | | No | | No | | No | | No | | Yes | | Yes | | Yes | | No | |
| StressCenter | iOS | NA | Disease management + education | Nil  USA | No | Yes | | Yes | | No | | Yes | | No | | No | | No | | Yes | | No | | Yes | | No | |

**Multimedia Appendix 2**: Characteristics of included apps (continuation)

| **App characteristics** | | | | | | | **Depression education modules content** | | | | | | | | | | | | | | | | | | | | |
| --- | --- | --- | --- | --- | --- | --- | --- | --- | --- | --- | --- | --- | --- | --- | --- | --- | --- | --- | --- | --- | --- | --- | --- | --- | --- | --- | --- |
| **App name** | **Platform** | **# of downloads** | **Type of**  **app** | **Developer team includes:**  **Country** | **Contains evidence-based information** | | **Sadness different from depression** | | **List depression symptoms** | | **Explain relapse/ recurrence** | | **Address stigma** | | **Diagnostic criteria** | | **Administer screening tool** | | **Explain treatment principles** | | **List treatment options** | | **Antidepress ants are non-addictive** | | **Lifestyle changes to improve symptoms** | | **Emergency help for suicidal users** |
| Teenagers With Experience | iOS | NA | Information & Education | Nil  UK | Yes | Yes | | Yes | | No | | Yes | | No | | No | | No | | No | | No | | Yes | | Yes | |
| UpLift - Depression & Anxiety | iOS | NA | Disease management + education | HCP  USA | Yes | Yes | | Yes | | No | | No | | No | | PHQ-9 | | No | | Yes | | No | | Yes | | Yes | |
| WellTrack | iOS | NA | Disease management + education | Nil  Canada | Yes | Yes | | Yes | | No | | No | | No | | DASS21 | | No | | Yes | | No | | No | | Yes | |
| What's Up? - A Mental Health App | iOS | NA | Disease management + education | HCP  Australia | Yes | No | | Yes | | No | | No | | No | | No | | No | | No | | No | | Yes | | Yes | |
| Youper | iOS | NA | Disease management + education | HCP  USA | Yes | Yes | | Yes | | No | | Yes | | No | | PHQ-9 | | No | | Yes | | No | | Yes | | Yes | |

HCP: Healthcare provider; Acad Inst: Academic Institution; Gov Ag: Government agency; MADRS: Montgomery-Asberg Depression Rating Scale
